# The Sufferings of Christ

**Published:** 1888-01-07

**Authors:** 


### Words of Consolation.

[Communications for this column should be addressed "Chaplain," care of the Editor of The Hospital, who will gratefully welcome any suggestions or inquiries from matrons, nurses, and the staff generally.]

### LXII.—The Sufferings of Christ.

*For Reading to the Sick.*

"And when they had platted a crown of thorns, they put it upon his head, and a reed in his right hand: and they bowed the knee before him, and mocked him, saying, Hail, King of the Jews."—(Matt. xxvii., 29.)

Early in the morning, before they led Him away to be crucified, the last cruelty perpetrated in the city was the mockery and derision of Christ as King. The hardened ruffians who had become exasperated by Pilate's vacillation, at last had matters entirely their own way. So before they laid the cross on the Sufferer, "they went through the whole heartless ceremony of a mock coronation, a mock investiture, a mock homage. In civilised nations all is done that can be done to spare every needless suffering to a man condemned to death; but among the Romans insult and derision were the customary preliminaries to the last agony. Around the brows of Jesus, in wanton mimicry of the Emperor's laurel, they twisted a green wreath of thorny leaves; in His tied and trembling hands they placed a reed for a sceptre; from His torn and bleeding shoulders they stripped the white robe with which Herod had mocked Him, which must now have been all soaked with blood, and flung on Him an old scarlet paludament—some cast-off war cloak, with its purple laticlave, from the Prætorian wardrobe. This they buckled over His right shoulder, and then each, with his derisive homage of bended knee—each with his infamous spitting—each with the blow over the head with the reed-sceptre, which his bound hands could not hold, they kept passing before Him with their mock salutation of 'Hail, King of the Jews.'" (Farrar.)

We cannot even imagine here what the King of Kings and Lord of Lords endured at having the deep sense of His own Majesty so awfully insulted and outraged. For the most part He thought little enough of Himself. In the midst of the sacrifice for the whole world, He could be so forgetful of self as to pay the minutest attention to His mother, to St. John, and to the penitent thief. But if He were truly Man, and King of mankind, and possessed therefore of the sense of dignity which every true man, and every true king amongst men, must possess, He could not even then die to such a feeling altogether. There is a wounding of this sense of dignity among the best of kings and queens, let us say rather in the very noblest natures, which is no wounding of pride. There is a dignity to be maintained in the midst of the most abject humiliation; and those who have the power to maintain it, and yet to bear meekly with provocation, perhaps enter most completely into the mind of Christ, as He stood robed in the purple garment, and crowned with thorns.

